# Oxidative Implications of Substituting a Conserved Cysteine Residue in Sugar Beet Phytoglobin BvPgb 1.2

**DOI:** 10.3390/antiox11081615

**Published:** 2022-08-20

**Authors:** Simon Christensen, Leonard Groth, Nélida Leiva-Eriksson, Maria Nyblom, Leif Bülow

**Affiliations:** 1 Pure and Applied Biochemistry, Department of Chemistry, Lund University, 22100 Lund, Sweden; 2 Biotechnology, Department of Chemistry, Lund University, 22100 Lund, Sweden; 3 Lund Protein Production Platform (LP3), Biology Building A, Lund University, 22362 Lund, Sweden

**Keywords:** phytoglobin, hexacoordination, crystallization, thermal stability, autoxidation, peroxidase activity, heme loss

## Abstract

Phytoglobins (Pgbs) are plant-originating heme proteins of the globin superfamily with varying degrees of hexacoordination. Pgbs have a conserved cysteine residue, the role of which is poorly understood. In this paper, we investigated the functional and structural role of cysteine in BvPgb1.2, a Class 1 Pgb from sugar beet (*Beta vulgaris*), by constructing an alanine-substituted mutant (Cys86Ala). The substitution had little impact on structure, dimerization, and heme loss as determined by X-ray crystallography, size-exclusion chromatography, and an apomyoglobin-based heme-loss assay, respectively. The substitution significantly affected other important biochemical properties. The autoxidation rate increased 16.7- and 14.4-fold for the mutant versus the native protein at 25 °C and 37 °C, respectively. Thermal stability similarly increased for the mutant by ~2.5 °C as measured by nano-differential scanning fluorimetry. Monitoring peroxidase activity over 7 days showed a 60% activity decrease in the native protein, from 33.7 to 20.2 U/mg protein. When comparing the two proteins, the mutant displayed a remarkable enzymatic stability as activity remained relatively constant throughout, albeit at a lower level, ~12 U/mg protein. This suggests that cysteine plays an important role in BvPgb1.2 function and stability, despite having seemingly little effect on its tertiary and quaternary structure.

## 1. Introduction

Hemoglobins (Hbs) are often oligomeric proteins belonging to the globin superfamily [1] found across all kingdoms of life [2]. Each Hb monomer displays the myoglobin-fold (Mb-fold) [3] consisting of six to eight α-helices labeled A–H. A binding pocket is formed between the opposing E and F chains. A proximal histidine residue on the F chain binds to the fifth coordination site of the heme b prosthetic groups central iron ion [4]. On the E chain a distal histidine residue fine-tunes binding of ligands, such as oxygen (O_2_), to the iron’s sixth coordination site [4,5,6,7,8]. Evolutionarily, however, the familiar role of O_2_ transport in blood-borne Hbs is a relatively new addition to its functional repertoire [6,8]. In plants, for example, phytoglobins (Pgbs), previously called nonsymbiotic Hbs [9], regularly engage in tasks unrelated to O_2_ transport [6,8]. This is because the distal histidine in Pgbs can reversibly occupy the sixth coordination site of iron. Pgbs, thus, exist in a configurational equilibrium between the penta- and hexacoordinated states [6,7,10].

The hexacoordination equilibrium constant (K_H_) of Pgbs correlates well with the three different phylogenetically classes (Class 1–3) [6]. K_H_ is the binding constant of the distal histidine, where a smaller number corresponds to a weaker hexacoordination. This allows for the equilibrium between the penta- and hexacoordinated states [11,12]. Class 1 Pgbs are distinguished by a weak hexacoordination (K_H_ ≈ 2) compared to Class 2 Pgbs (K_H_ ≈ 100). Hexacoordination impacts O_2_ interactions, supported by the averaged differences seen in O_2_ affinities (p_50_ ≈ 2 nM and p_50_ ≈ 340 nM) association rates (~25 μM^−1^·s^−1^ and ~1 μM^−1^·s^−1^) and O_2_ dissociation rates (~0.16 s^−1^ and ~1 s^−1^) for Class 1 and 2 Pgbs, respectively [6]. Thus, Class 1 Pgbs are poorly suited for O_2_ transport, which is apparent due to their low O_2_ dissociation rate. Instead, phytoglobins can be viewed as O_2_ carriers, whereby class 1 Pgbs are critical components in electron transport due to their high O_2_ affinity and redox potential, thus allowing maintenance of the energy status during O_2_ deprivation [13,14].

Due to the very high O_2_ avidity of Class 1 Pgbs, these proteins generate a tight and slow binding of O_2_ well suited for O_2_-dependent nitric oxide (NO) scavenging in low O_2_-containing environments [2,8,12,13,14,15]. In general, for Pgbs to act as a NO scavenger, the protein must bind O_2_ first, followed by the incorporation of NO. However, the dissociation of O_2_ from the heme may ultimately lead to autoxidation, resulting in forming the ferric state (Fe^III^) and a superoxide radical (O_2_^•−^) [16]. Thus, analysis of autoxidation rates are particularly relevant for globins because they are much less reactive in the ferric state and unable to bind O_2_, than in the ferrous (Fe^II^) state [7].

Plants ubiquitously express one or more of the three Pgb classes, with some plants expressing all [6,14]. This was observed in a previous expression-pattern study of sugar beet where four new Pgbs (BvPgb1.1, BvPgb1.2, BvPgb2, and BvPgb3) were identified. Interestingly, BvPgb1.2 was found to be predominantly expressed in the seeds of sugar beets [17], suggesting that Pgbs can play important roles in plant development and germination. The biophysical properties of these sugar beet Pgbs were further characterized in a recent follow-up study [18]. This investigation concluded that, even if the Pgbs have intrinsic properties to carry out certain enzymatic reactions, the location and concentration of ligands, such as O_2_, NO, and nitrite (NO_2_), will ultimately determine the protein activity [18]. This challenges the notion that Pgbs primarily serve as homeostatic energy agents during O_2_ deprivation, as previously mentioned. However, to date, neither their three-dimensional structure nor the overall biophysical properties of BvPgbs have been elucidated.

Hbs have a remarkable sequence diversity, where some proteins share less than 20% identity [19,20]. Despite this variation, some residues still display varying degrees of conservation [21]. Cysteine residues, for example, appear in Hbs with varying abundance in functionally important places [22]. The precise function has been under investigation for a long time. In human Hb for instance, Cys93 in the β-chain has been hypothesized to be important for the subunits to stoichiometrically combine into a functional protein [23].

Furthermore, oxidation of the same residue is dependent on its proximity to the heme group or the protein–solvent interface [24]. Most Pgbs, including BvPgb1.2, have a single conserved cysteine residue on the E-chain [22,25]. In barley, this cysteine is found in position 79. It was concluded that the Cys79 residue was indirectly involved in metHb reduction and NO turnover [26]. In addition, a Cys-mutant in barley Hb was more prone to be present in a monomeric form, instead of the usual homodimer. Thus, the lack of disulfide bridge formation between the cysteine residues in each monomer led to destabilization of the quaternary structure and less protection of the ferrous state inside the heme pocket [22].

In this comparative study, we investigated the role of Cys86 in BvPgb1.2 by generating an alanine-substituted mutant. The imposed substitution did not affect the overall tertiary structure, but led to significantly faster autoxidation rates and a minor increase in thermal stability. Furthermore, the substitution did not affect the degree of dimerization and revealed essentially no heme loss for either WT or mutant. However, hemoglobins in general often harbor intrinsic peroxidase-like activities, which can cause oxidative damages to surrounding biomolecules such as lipids, proteins, and nucleic acids. Regulation of peroxidase activity is, hence, essential in practical applications of these proteins. Therefore, we analyzed such enzymatic properties of the WT and substituted mutant by a simple in vitro assay. Taken together, this work provides new knowledge regarding the oxidative features of Class 1 Pgbs and highlights the intrinsic effects of the conserved cysteine on a structural–functional level.

## 2. Materials and Methods

### 2.1. Recombinant Protein Production

Wildtype (WT) BvPgb1.2 (GenBank: KF549981) was expressed in BL21-DE3 *Escherichia coli* using a pET-DEST42 vector as described previously [18]. Site-directed mutagenesis of Cys86 (TGT) to Ala86 (GCC) was carried out by PCR amplification using Phusion^TM^ High-Fidelity PCR mix (Thermo Scientific^TM^) supplemented with 0.6 µL of 100% DMSO. A forward primer consisting of 5′–ATG AGC GTT TTT GTC ATG ACC **GCC** AAA AGC GCA GCT CAG CTG CGC–3′ together with a reverse primer 5′–GCG CAG CTG AGC TGC GCT TTT **GGC** GGT CAT GAC AAA AAC GCT CAT–3′ was used to achieve the desired Cys86Ala mutant. The mutation was confirmed by sequencing of several positive clones.

### 2.2. Cultivation of Protein-Expressing Cells

The expression of both BvPgb 1.2 WT and Cys86Ala was conducted using previously described methods [18].

### 2.3. Protein Purification

BvPgb1.2 WT and Cys86Ala expressing cells were treated and purified according to earlier published methods [18], but the Q-Sepharose HP step was omitted. The final BvPgb1.2 fractions had an absorbance ratio at 412 nm and 280 nm (A_412_/A_280_) in the range 2.5–3, corresponding to >95% purity. BvPgb1.2 fractions were then concentrated using 10 kDa Vivaspin^®^ 20 mL ultrafiltration units (Vivascience) and stored at −80 °C.

### 2.4. Protein Crystallization

To prepare the cyanide form of BvPgb1.2 WT and Cys86Ala, the purified proteins were dialyzed in a solution containing 10 mM potassium ferricyanide and 1 mM potassium cyanide dissolved in 50 mM Tris-HCl pH 8.5 for 8 h in a 0.5 L solution. This process was performed twice. The cyanide-modified protein solution was passed through a PD10 column (Cytiva Life Science, Uppsala. Sweden) to remove excess cyanide and the protein was then concentrated using 10 kDa Vivaspin^®^ 20 mL ultrafiltration units (Vivascience).

The crystallization of the WT and alanine substituted protein was achieved under slightly different conditions. For the WT protein, the sample was concentrated to 35 mg/mL in 50 mM Tris-HCl pH 8.5. Screening against commercially available screens was performed with a Mosquito crystallization robot (SPT Labtech) using drops with total volume of 300 nL and a 1:2 ratio of protein sample to reservoir solution. Crystals appeared after 1 week of incubation in 20% (*w*/*v*) PEG 6000, 0.1 M citrate buffer at pH 5.0 and 20 °C, and they reached full size (50 × 200 × 200 µm) within 20 days. The crystallization procedure was slightly modified for the cyanide-treated Cys86Ala mutant. The sample was concentrated to 60 mg/mL in 50 mM Tris-HCl pH 8.5), but diluted to 30 mg/mL with 50 mM Tris-HCl pH 6.8 prior to crystallization trials.

Screening was performed using drops with total volume of 300 nL and ratio 2:1 protein sample to reservoir solution. The crystals appeared after 1 day of incubation in 25% (*w*/*v*) PEG 1500, 0.1 M MIB (sodium malonate dibasic monohydrate, imidazole, boric acid, molar ratios 2:3:3; molecular dimension) at pH 8.0 and 20 °C, and they reached full size (25 × 300 × 800 µm) within 14 days.

Crystals were harvested and briefly cryoprotected in a reservoir solution containing 20% glycerol before being flash frozen in liquid nitrogen. Testing for diffraction and data collection were conducted at the BioMAX beamline at the MAX IV Laboratory (Lund, Sweden).

Diffraction data were collected by fine-slicing with an oscillation range of 0.1° to 360° total. The data were processed with AutoPROC (Global Phasing) [27] to a resolution of 1.9 Å and 2.2 Å, respectively, for the WT and mutant protein. The structure was solved by molecular replacement with PHASER of the PHENIX software suite [28], using PDB entry 3zhw.pdb as search model. Electron and difference density maps were manually inspected; the model was improved using Coot [29] and several rounds of refinement with PHENIX. The calculation of Rfree used 5.25% and 5.12% of the data for WT and Cys86Ala, respectively. The PDB entries for WT and Cys86Ala are 7ZOS and 7Z1U, respectively. Statistics for data and refinement can be seen in Supplementary Tables S1 and S2.

### 2.5. Size-Exclusion Chromatography

To examine possible effects of the Cys86 mutation on the oligomeric assembly, purified BvPgbs were diluted with 50 mM NaP containing 150 mM NaCl pH 7.0 to 1 mg/mL (5.2 µM) solutions. Analytical size-exclusion chromatography (SEC) was carried out with a Superdex 7510/300 GL column (CV = 24 mL) on an ÄKTA™ Avant system (Cytiva Life Science, Uppsala, Sweden) calibrated with conalbumin (75 kDa), carbonic anhydrase (29 kDa), ribonuclease A (13.7 kDa), and aprotinin (6.512 kDa) as molecular weight (M_w_) standards (Cytiva Life Science). All protein solutions were run in volumes of 100 µL with a protein concentration of 1 mg/mL. The M_w_ of the BvPbg solutions was estimated by fitting the measured retention volume (V_r_) to a semi-log plotted calibration curve (Supplementary Figure S1).

### 2.6. Autoxidation Kinetics

To assess the influence of Cys86 on autoxidation, 10 µM oxygenated BvPgb samples in 1× PBS were incubated separately in sealed cuvettes at 25 °C or at 37 °C for 72 h. Absorbance spectra between 300 and 700 nm were recorded using a Cary 60 UV/Vis system (Agilent Technologies) every 15 or 10 min for the 25 °C and 37 °C incubations, respectively. The change in absorbance at 576 nm (A_576_) due to oxidation of ferrous to ferric BvPgbs was monitored and analyzed using XPFit (SoftScientific). This curve-fitting solved for the autoxidation rate constant (λ) by acquiring the mean lifetime (τ) for both WT and Cys86Ala BvPgb1.2. Half-life (t_1/2_) was used as a measure of autoxidative tendency. The model used the following models:

N(t) = N(0) × e^−^^λt^, (1)

λ = 1/τ,(2)

t_1/2_ = ln (2)/λ, (3)

where λ is the decay rate constant (autoxidation rate), τ is the mean lifetime (also called the scaling time), and t_1/2_ is the half-life, i.e., the time required to fall to half of its initial value.

Experiments were repeated three times (*n* = 3), and standard deviations were estimated in Microsoft Excel.

### 2.7. Thermal Stability

To investigate the effect of Cys86 on structural thermal stability, 26 µM oxygenated protein solutions in either 1× PBS or 10× PBS were incubated in 96-well plates (Sarstedt) at 25 °C and 37 °C for 7 days. Thermal stability was analyzed through nano-differential scanning fluorimetry (nanoDSF) using a Prometheus NT.48 instrument containing aggregation optics (NanoTemper Technologies). Eight replicates of ~10 μL samples were taken daily and loaded onto capillaries (NanoTemper Technologies). The LED intensity for excitation at 280 nm was set to 40%. Sample fluorescence intensities at 350 nm (F_350_) and 330 nm (F_330_) were recorded whilst running a temperature ramp from 20 to 95 °C with 1 °C/min increments. The melting point (T_m_) for the BvPgb solutions was acquired by a first-order derivative of the F_350_/F_330_ fluorescence ratio plotted against the temperature. An example of the transition state determination by the Prothetheus software can be seen in Supplementary Figure S5. The experiments were repeated three or more times (*n* > 3) and standard deviations were estimated in Microsoft Excel.

### 2.8. Heme-Loss Assay

Heme loss from BvPgb WT and Cys86Ala was determined using the heme scavenger apomyoglobin H64Y/V67F (ApoMb) according to the method developed by Hargrove et al. [30] and Silkstone et al. [31]. Both proteins were converted to the ferric form using potassium ferricyanide in excess. The proteins were then incubated at 25 °C and 37 °C, respectively, for up to 20 h in 100 mM NaP pH 7.2 supplemented with 150 mM sucrose together with ApoMb in excess. The final concentrations of proteins were 6.25 µM and 30 µM of BvPgbs and ApoMb, respectively. The reactions were monitored by scanning the spectra of the BvPgbs in the 250–700 nm range using a Cary60 UV/Vis spectrophotometer (Agilent Technologies). The heme transfer could be followed at 600 nm, where the characteristic spectrum of ApoMb could be distinguished. Wildtype fetal Hb (HbF) in the ferric from was used as a positive control for the same conditions [32]. The experiments were repeated three times (*n* = 3).

### 2.9. Peroxidase Activity Measurements

An assessment of the peroxidase activity for the studied proteins was based on previous reported results by Kvist et al. [33]. The formation of the 2,2′-azino-di-(3-ethylbenzthiazoline sulfonic acid) diammonium salt (ABTS) (Sigma) radical cation was monitored at 415 nm and converted into concentration of product using ε_415_ = 36 mM^−1^·cm^−1^. Each reaction was carried out in 50 mM Tris-HCl buffer pH 8.5 together with 1 µM BvPgb, 10 mM H_2_O_2_, and 5 mM ABTS, and the reaction was monitored using a Cary60 UV/Vis spectrophotometer (Agilent Technologies) at 415 nm. The BvPgbs were incubated at 22 or 37 °C, and the absorbance was measured after 0, 0.5, 1, 4, and 8 h for the first day, followed by one single measurement per day for 7 days in total. Corrections were made for the minor background activity of the assay observed without any added proteins. The experiments were repeated three times (*n* = 3), and standard deviations were estimated in Microsoft Excel.

## 3. Results

### 3.1. Protein Expression and Purification

According to a previously described protocol, the WT and the Cys86Ala mutant were successfully expressed in *E. coli* [18]. However, the final yield of the mutant was approximately 27% lower than the WT. The same method could be used during the following protein purification steps involving two chromatographic steps, ion exchange and hydrophobic interaction chromatography. Both proteins behaved identically on these resins, indicating that no surface modification had occurred due to the substitution. After these steps, the proteins were more than 95% pure.

**Figure 1:**
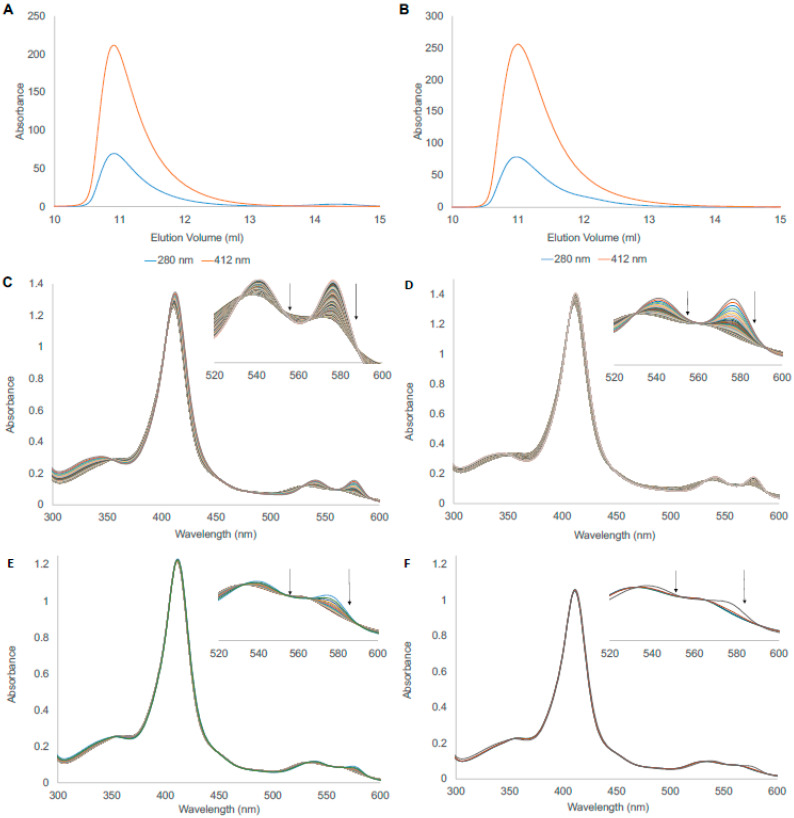
Size-exclusion chromatography and autoxidation measurements of BvPgb1.2 WT and Cys86Ala. (**A**,**B**) BvPgb1.2 WT and Cys86Ala, respectively. Data form 280 nm (protein backbone absorption) and 412 nm (heme-containing proteins) are presented. Both species eluted ~10.9 mL, corresponding to a molecular weight ~40 kDa (Supplementary Figure S1). (**C**,**D**) Complete autoxidation spectra for BvPgb1.2 WT 25 °C and WT at 37 °C, respectively. (**E**,**F**) Complete autoxidation spectra for BvPgb1.2 Cys86Ala 25 °C and Cys86Ala at 37 °C. For the autoxidation, a zoomed-in view is presented in the 500–600 nm range for depiction of the oxidation process from the ferrous to ferric oxidation state (Fe^II^→Fe^III^).

Since the trials in the purification developing phase suggested that the BvPgb1.2 may be present in a dimeric form, an analytical size-exclusion chromatography (SEC) was performed to more accurately assess the potential degree of oligomerization (Figure 1). This revealed identical chromatographic profiles for both WT and Cys86Ala BvPgb1.2 (Panel 1 and 2, respectively), with V_r_ ≈ 10.9 mL corresponding to a M_W_ ≈ 40 kDa, which compares well with the theoretical M_W_ = 38.4 kDa of a dimeric BvPgb1.2. When analyzed further in the obtained chromatograms, the degree of dimerization was >99% for both proteins. Equine myoglobin (Mb), a monomeric protein, was used as a positive control with V_r_ ≈ 12.8 mL, corresponding to a M_W_ ≈ 19 kDa. This indicates that Cys86 did not impact the dimeric assembly of BvPgb1.2 under the conditions used. However, minor differences in intensity at the Soret peak at 412 nm between the WT and mutant, probably due to the increased oxidation rate, suggest that Cys86 partly influenced the microenvironment of the heme moiety.

### 3.2. Autoxidation

The dissociation of O_2_ from the heme may ultimately lead to autoxidation, resulting in the formation of the ferric state (Fe^III^) and superoxide (O_2_^•−^) [16]. Thus, important insights regarding redox stability can be obtained by examining the autoxidation rates for the WT and the mutant. The initial spectra for the complete autoxidation of BvPgb 1.2 WT and Cys86Ala can be seen in Figure 1, where panels (C) and (D) correspond to the WT at 25 and 37 °C, respectively. In contrast, panels (E) and (F) correspond to the mutant at the same temperatures. Here, changes in autoxidation rates were detected at both temperatures and protein samples. The changes became more evident when the area between 520 and 600 nm was more closely examined. The wavelengths where the transition was most well defined from these spectra were observed at 542 and 576 nm. The exponential decay rate was estimated by comparing the decrease in absorbance at 576 nm during the time course studied (Supplementary Figure S2).

When the exponential decay model was used in XPFit, mean lifetime (τ), autoxidation rate (λ), and half-life (t_1/2_) were obtained for the WT and Cys86Ala proteins at the different temperatures (Table 1). Here, the mutant had a 16.7× higher autoxidation rate than the WT at 25 °C. At 37 °C, the mutant oxidized ~14.4× faster than the WT. However, when analyzed at these two different temperatures, WT at 37 °C and Cys86Ala at 25 °C showed similar behaviors, especially in terms of mean lifetime, autoxidation rate, and half-life.

### 3.3. Structural Comparison

**Figure 2:**
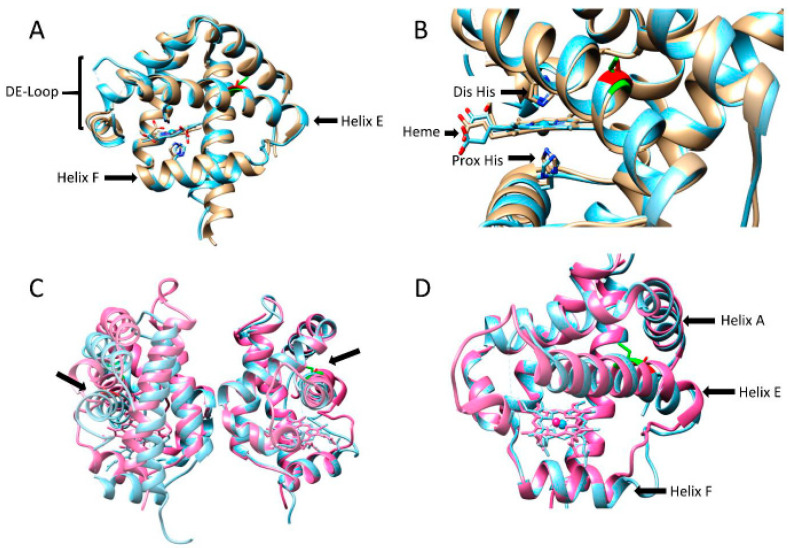
Crystal structure comparison of BvPgb 1.2 WT, Cys86Ala, and Barley Hb. (**A**) Structure comparison between BvPgb 1.2 WT (gray) and Cys86Ala (light blue), highlighting helices E and F in addition to the missing DE-loop. (**B**) Zoomed-in view of the heme-group, displaying the heme group, proximal and distal histidinem and the orientation of the cysteine (green) and alanine (red) in the WT and mutant, respectively. (**C**,**D**) Comparison between Cys86Ala (light blue) and Barley Hb (PDBID: 2OIF) (pink), showing orientation of the cysteine in Barley Hb (green) and BvPgb 1.2 Cys86Ala (red).

After purification, the two phytoglobins, WT and Cys86Ala, were successfully crystallized. The conditions for crystallization differed slightly, indicating minor differences in the surface properties of the two proteins. However, the overall 3D structures were very similar (Figure 2). The main differences can be found in the helical arrangement of the F-helix. In the WT, this helix starts with a Glu residue, while, in the mutant, the helix seems shorter and starts two positions downstream with a Ser residue. As a result, the direction of the side-chains has different orientations as clearly observed in the proximal His, which is almost in a perpendicular position compared to the proximal His in the WT.

A previous report regarding Pgb from barley showed that the conserved Cys residue greatly influences the dimerization of the protein. In WT, less than 5% of the protein population was present as a monomer, while a Cys substituted mutant exhibited 30–50% monomer content [22]. Furthermore, an intermolecular disulfide bond between the cysteine residues in each monomer was believed to be an important stabilizer of the tertiary and quaternary structure of this Pgb. Even though this possibility cannot be dismissed for BvPgb1.2, it is evident that the substituted cysteine did not contribute to increased monomer content. The monomers in the dimers showed antiparallel orientation, with the heme groups at the opposite ends of the dimers. In Figure 2A,B, the position of the Cys in WT and Ala in the mutant can be seen in green and red, respectively. In addition, a similar comparison between barley Hb and Cys86Ala can be seen Figure 2C,D. The side-chains of the Cys residues are facing outward from the protein surface, and not into the interface between the subunits. This is opposed to previous reasoning where a disulfide bridge was supposed to stabilize the dimer [22].

If the Cys substitution caused a difference in the F-helix between WT and Cys86Ala, then it was not evident from the elucidated structure. For most Class 1 Pgbs, the E-helix is bent toward the center of the porphyrin ring due to direct coordination of the distal histidine with the heme iron [25,34]. In helix E, where the mutation was located, no apparent differences could be observed compared to the helix E in the WT.

### 3.4. Thermal Stability of BvHb1.2 WT and Cys86Ala

**Figure 3:**
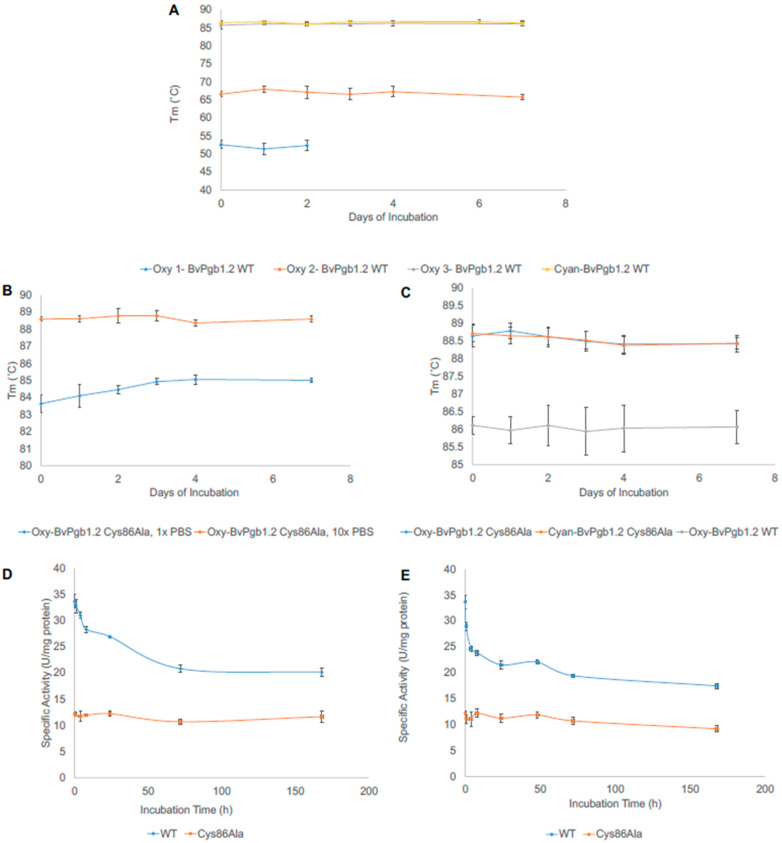
Thermal stability and peroxide activity measurements for BvPgb 1.2 and Cys86Ala. (**A**) Stability measurement for oxy- and cyanide BvPgb1.2 WT in 10× PBS at 25 °C during the 7 day incubation period. Three different transition states were detected (oxy 1, 2, and 3) for the first 2 days. The transition states for oxy 2 and 3 and cyanide was detected throughout the incubation period. (**B**) Stability measurement for oxy-BvPgb1.2 Cys86Ala in 1× and 10× PBS at 25 °C during the 7 day incubation period. (**C**) Stability measurement for cyanide- and oxy-Cys86Ala and oxy-BvPgb1.2 in 10× PBS at 37 °C during the 7 day incubation period. (**D**,**E**) Peroxidase activity for WT and Cys86Ala at 22 °C and 37 °C, respectively. The measurements were conducted in triplicate (*n* = 3), and statistical significance (*p* < 0.05) was determined using an unpaired *t*-test.

The thermal stability indicates if the unique structure and functionality of a protein can also be maintained at elevated temperatures. Upon heating, phytoglobins can pass several transitions linked to oligomerization changes, packing of helices, and interaction between the heme and the apoglobin. In the case of oxy-BvPgb1.2 WT, three different temperatures were determined to show a transition state (53, 67, and 86 °C) (Figure 3A).

The thermal stability of the proteins was also examined after an extended time of incubation at 25 and 37 °C. After 2 days of incubation at 25 °C, the first transition state (oxy 1) disappeared. The two remaining transition states were stably maintained during the entire incubation period of 7 days. The cyanide form of the protein provided more structural stability, and the two lower transition states could not be seen in the cyanide-bound WT protein (Figure 3A) or in Cys86Ala (Figure 3B).

Another critical aspect to consider was how the protein would respond at physiological temperature (37 °C), since several potential practical applications of the proteins are found at this temperature (Figure 3C). The same conditions were also investigated for cyanide- and oxy-Cys86Ala at 37 °C, but no differences were seen. As mentioned before, only one transition state could be detected for the mutant’s oxy- and cyanide form.

### 3.5. Peroxidase Activity

Pseudoperoxidase activity is one of the general characteristics of Hbs. The main point of this activity is the transformation of ferrous (Fe^II^) to highly reactive intermediates. In this reaction, the oxidant H_2_O_2_ drives a catalytic cycle that includes two intial steps: (1) initial oxidation of Fe^II^ to ferryl (Fe^IV^), and (2) autoreduction of the ferryl intermediate to ferric (Fe^III^) (met). Using additional H_2_O_2_, the ferric form can be converted into a ferryl intermediate. This oxidative state may be utilized for the peroxide activity measurements, which is often monitored using a substrate forming an easily monitorable product that can be detected by UV/Vis spectrophotometry [33,35]:

Heme-Fe^III^ + H_2_O_2_ → Heme*^+^-Fe^IV^ = O + H_2_O. (4)

Heme^•+^-Fe^IV^ = O + AH → Heme-Fe^IV^ = O + A^•^ + H^+^. (5)

Heme-Fe^IV^ = O + AH + H^+^ → Heme-Fe^III^ + A^•^ + H_2_O. (6)

By measuring the absorbance of the radical product (A^•^), the peroxidase activity can be estimated. The detectable radical formation of ABTS was used in this study to quantify this. The stability of the proteins was estimated by measuring the activities of WT and Cys86Ala over a more extended period, up to 7 days. The first tests were made by incubating the proteins at 22 °C (Figure 3D). The WT showed almost three times the initial activity compared to Cys86Ala (33.7 versus 11.7 U/mg protein). Upon incubation, the WT gradually lost its activity, going from the initial value to 20.2 U/mg protein. In contrast, even though the mutant had a lower initial reactivity, this was maintained throughout the incubation time.

The same pattern was observed at 37 °C (Figure 3E), albeit with a faster decline in peroxidase activity for the WT, reaching a final activity slightly lower than at 22 °C (17.4 U/mg protein). However, the activity of the mutant was stable during the entire incubation period; however, as with the WT, it reached a lower final activity in comparison with the lower temperature (9.2 U/mg protein). Thus, the removal of the conserved cysteine significantly affected the reactivity of the protein.

### 3.6. Heme Loss from WT and Cys86Ala

**Figure 4:**
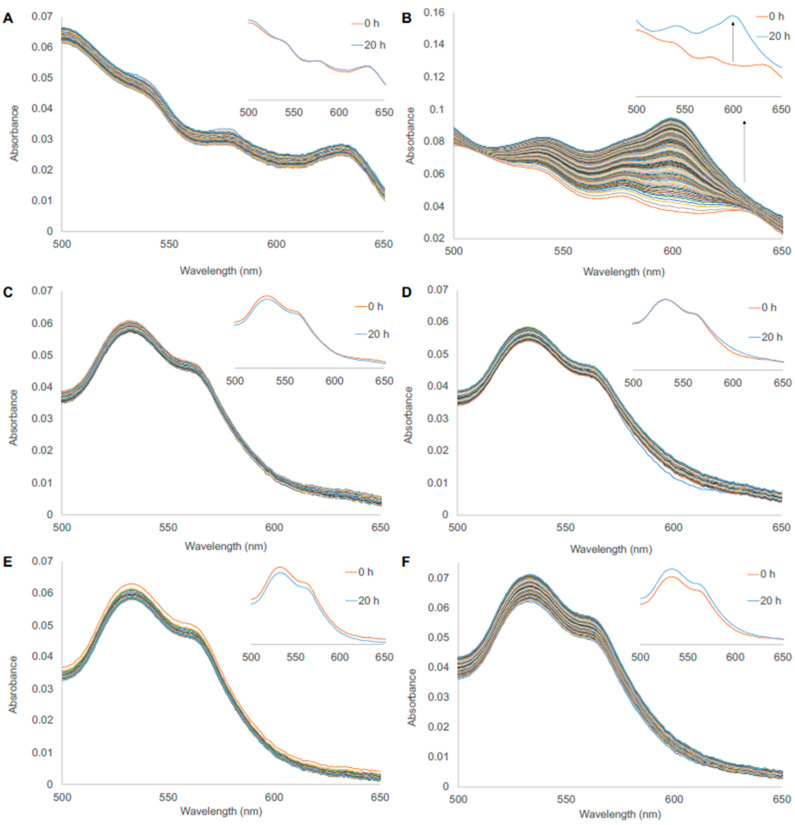
Heme-loss assay using ApoMb as a heme-scavenger. (**A**,**B**) Fetal hemoglobin (HbF) blank sample and addition of ApoMb, respectively. The blank sample retained the initial spectrum after 20 h, while the Mb spectrum was detected in the right panel, most predominately seen as an increase in absorbance at 600 nm. (**C**,**D**) BvPgb1.2 WT blank sample and addition of ApoMb, respectively. In both cases, the initial spectra remained constant, i.e., no Mb spectrum at 600 nm could be detected. (**E**,**F**) BvPgb1.2 Cys86Ala blank sample and addition of ApoMb, respectively. In both cases, the initial spectra remained constant, i.e., no Mb spectrum at 600 nm could be detected. All experiments were conducted at 25 °C; spectra were measured in 10 min intervals for 20 h, and the ferric form of the Hbs was used.

In order to estimate the possibility of heme loss from the phytoglobins, the apomyoglobin (ApoMb) developed by Hargrove et al. [30] was used as a catcher protein for the free heme. Here, heme loss could be detected at 600 nm, where the holomyoglobin absorbs most prominently. To test the functionality of the ApoMb, a ferric human fetal Hb with known characteristics was used as a positive control [32]. The distinctive absorbance increase at 600 nm could be seen, showing a functional ApoMb (Figure 4A,B).

The heme-loss assay for BvPgb 1.2 WT and Cys86Ala mutant is shown in Figure 4. Only minimal heme loss could be detected when compared to the HbF positive control (panel A and B) for both WT (panel C and D) and Cys86Ala (panel E and F) at 25 °C. The increase at 600 nm seen for the phytoglobins was, thus, marginal compared to the positive control, indicating much less tendency to release the heme group for the BvPgbs. When comparing the WT and Cys86Ala proteins, no difference was observed. The same pattern was seen at 37 °C (Supplementary Figure S3). Thus, both phytoglobins showed a very high ability to retain the heme group, even under scavenging conditions.

## 4. Discussion

Plant-based hemoglobins, such as leghemoglobins and phytoglobins, have often been suggested to be valuable ingredients in meat-like alternative food products to provide taste and color. Over the years, the biophysical properties of myoglobin present in meat have been carefully examined [36]. However, much less is known about hemoglobins of plant origins. Several intrinsic properties need to be evaluated, and they may be engineered to generate globin alternatives that are safer for human consumption. Heme loss, thermal stability, and peroxidase and redox activities are essential biophysical parameters that need to be fully understood when the proteins are to be used in a context outside their natural environment. For instance, it is particularly important to control and optimize the processing parameters and the final product quality when such proteins are added as supplements to a novel food manufacture.

Proteins often carry conserved cysteine residues. These residues may play several different roles, such as stabilizers for maintaining the native protein structure. The thiol-containing side-chains are often buried in the interior of the polypeptide chains to allow disulfide bonds or, alternatively, located on the surface to stabilize oligo- or multimeric protein structures. Cysteine residues can also be part of an active center to promote reactivity. Sulfhydryl containing side-chains are powerful antioxidants and may act as redox hotspots in proteins. In hemoglobins, electrons are often transferred from the iron atom in the heme group to cysteine residues [31]. The cysteine acts as an electron recipient, but the sulfhydryl side chain can be reduced back to the native state if an antioxidant is present in the environment. This scenario requires that the Cys residue is located on the protein surface, close to the heme group. In the elucidated WT protein, the distance between the conserved cysteine and the heme iron was 17.3 Å (Supplementary Figure S4). This can in a first glance be viewed as remote. However, it is well within the 19 Å distance limit that enables electron transfer on ms timescale [37,38]. This may also indicate that the protein is dynamic in this region with a flexible helix E able to move closer to the heme iron. Alternatively, a relay mechanism has evolved involving other residues close to the heme. In particular, Tyr residues should be examined, since they are often involved in the redox activity of hemoglobins [39]. When the active center of BvPgb was investigated in more detail, one Tyr residue close to the heme group could easily be identified in position 115, with a distance of 9.24 Å from the heme iron (Supplementary Figure S4). Therefore, it is plausible that these two residues, Tyr 115 and Cys 86, are both involved in maintaining the redox stability of the iron.

Undoubtedly, the conserved cysteine present has a protective role against autoxidation and may be involved in direct electron transfer from the heme iron, affecting the oxidation state. The redox balance is altered when the cysteine is removed, reducing the ability of the heme to stay in its active ferrous form. Thus, since the oxidative state of Cys86Ala is pushed toward the inactive ferric state, fewer radicals are formed in the reaction with the peroxide, ultimately leading to less activation of the possible substrates in the peroxidase reaction. As mentioned previously, effects of the cysteine substitution in Pgb from barley have previously been examined, where enhanced autoxidation was one of the aspects. A 1000-fold increase in oxidation rate for the mutant was reported [22]. Even though a significant increase in autoxidation for the BvPgb1.2 mutant could be seen, it had considerably less impact than in barley Hb. This could be due to the different orientations of the cysteine between barley (monocot) and sugar beet (eudicot) (Figure 2) and the resulting change in structural integrity in the barley hemoglobin [40], which was not seen in the our Cys86Ala mutant.

Another essential parameter is the thermostability of the two proteins. The T_m_:s in Figure 3 shows that the mutant had a melting point approximately 2.5 °C higher than the WT at both temperatures. However, the three transitions states found for the WT were absent in the mutant under the same conditions. Thus, these results are in line with the oxidation findings, pointing to the fact that the WT is more prone to function during heat stress for long periods.

According to previous melting temperatures for a broad span of proteins, a thermostable protein is considered to have a T_m_ > 65 °C [41]. In the case of myoglobin, different melting temperatures have been observed, ranging from apoMb from *Aplysia limacine* (T_m_ = 52 °C) [42] to horse heart Mb (76 °C) [43]. Furthermore, human neuroglobin is very thermally robust (T_m_ = 100 °C) [41,44]. Therefore, the observed melting temperature of BvPgb1.2 (and the mutant) suggests a thermally stable protein, and it is the first Pbg to be characterized in this way. The explanation for this might be the high degree of hexacoordination reported for Pgb [44]. However, simulations have also shown that the flexibility of the CD region could contribute to the thermal stability in the globin family [45,46].

## 5. Conclusions

This study investigated the biochemical effects of substituting the conserved cysteine residue in the phytoglobin BvPgb1.2. Both WT and Cys86Ala were characterized by protein structural determination, degree of dimerization, thermal stability, peroxidase activity, and autoxidation rate. The mutation did not affect the degree of dimerization but enhanced the oxidizing rate and improved thermal stability. Moreover, the crystal structure revealed the orientation of the cysteine residue and suggested a protective role against oxidation, but to a different degree from previously published results. In addition, both proteins showed minimal degree of heme loss. The peroxidase activity for the WT was approximately 2–3× higher than that for the mutant. The removal of the cysteine provided an altered redox environment of the heme iron, which affected all investigated features to a substantial degree. This work provides comprehensive knowledge of the intrinsic WT globin properties, which should promote its further biotechnological use.

## 6. Patents

S.C., L.G., and L.B. have a pending patent application relating to the BvPgb 1.2 Cys86Ala mutant and its related characteristics.

## Figures and Tables

**Table 1:** Summary of autoxidation parameters for the WT and Cys86Ala using the exponential decay model.

	N_0_	λ (h^−1^)	τ (h)	t_1/2_ (h)
WT 25 °C	0.096 ± 0.002	0.014 ± 0.001	72.2 ± 0.180	50.1 + 0.125
WT 37 °C	0.082 ± 0.005	0.214 ± 0.003	4.68 + 0.069	3.24 + 0.005
Cys86Ala 25 °C	0.043 ± 0.005	0.234 ± 0.012	4.28 + 0.226	2.97 + 0.157
Cys86Ala 37 °C	0.035 ± 0.003	3.08 ± 0.035	0.342 + 0.004	0.225 + 0.003

## Data Availability

Data are contained within the article and Supplementary Materials.

## Supplementary Materials

The following supporting information can be downloaded at www.mdpi.com/article/10.3390/antiox11081615/s1: Figure S1. Standard curve from size-exclusion chromatography; Figure S2. Exponential decay spectra for BvPgb1.2 WT and Cys86Ala mutant at 540 and 576 nm as a function of time; Figure S3. Heme-loss assay using ApoMb as heme-scavenger at 37 °C; Figure S4. Distance determination between heme and surrounding tyrosine and cysteine residue in BvPgb 1.2 WT; Figure S5. Thermal stability determination using Prometheus NT.48 (NanoTemper Technologies); Table S1. Crystallographic table of data used in structure determination of BvPgb 1.2 WT and final model statistics; Table S2. Crystallographic table of data used in structure determination of BvPgb 1.2 Cys86Ala and final model statistics.
