# Supplementary material for: Oxidative Implications of Substituting a Conserved Cysteine Residue in Sugar Beet Phytoglobin BvPgb 1.2
Source: Antioxidants (Basel). 2022 Aug 20;11(8):1615. doi: 10.3390/antiox11081615 (PMC9404779; doi:10.3390/antiox11081615)
Supplement: Supplementary file 1 [file antioxidants-11-01615-s001.zip › antioxidants-1816760-supplementary.pdf]

Supplementary Material

Crystallographic table for both BvPgb 1.2 WT and Cys86Ala are shown in Table S1 and Table S2, respectively. The final structures for these proteins can be accessed though the Protein Data Bank with PDB-ID 7ZOS (WT) and 7Z1U (Cys86Ala).

Table S1. Crystallographic table over data used in structure determination of BvPgb 1.2 WT and final model statistics.

**Data collection**	**BvPgb1.2 WT (PDB ID 7ZOS)**
Space group	P4212
Cell dimensions<br>a, b, c (Å)	96.75 96.75 47.90
α, β, γ (°)	90.0 90.0 90.0
Wavelength (Å)	0.95372
Resolution (Å)	68.413- 1.901 (1.934- 1.901)*
Rmerge	0.083 (1.664)*
Number of observations	471282 (23568)*
Number of unique observations	18443 (907)*
Mean I/σ(I)	23.7 (2.5)*
Completeness (%)	100.0 (100.0)*
Multiplicity	25.6 (26.0)*
CC(1/2)	0.999 (0.727)*
**Refinement**	
Resolution (Å)	47.900- 1.901
Number of reflections	18440
R_{work}/R_{free}	0.2027 / 0.2353
R_{free} test set, reflections (%)	5.25
No. of non-H atoms	1298
Ligands	1 heme, 1 CN^-, 1 $[Fe(CN)6]^{-4}$
R.m.s. deviation from ideal geometry	
Bonds (Å)	0.010
Angles (°)	1.465
Average B, all atoms (Å^2)	42.94 (range 24.77– 90.03)
Ramachandran plot	
Favoured regions (%)	100.00
Allowed regions (%)	0
Outliers (%)	0
MolProbity clash score	2.91

* Outer resolution shell

Table S2. Crystallographic table over data used in structure determination of BvPgb 1.2 Cys86Ala and final model statistics.

**Data collection**	**BvPgb1.2 Cys86Ala (PDB ID 7Z1U)**
Space group	P1 21 1
Cell dimensions<br>a, b, c (Å)	36.85 73.92 62.83
α, β, γ (°)	90.00 105.05 90.00
Wavelength (Å)	0.97996
Resolution (Å)	60.675 - 2.242 (2.281 - 2.242)*
Rmerge	0.072 (0.874)*
Number of observations	109719 (5545)*
Number of unique observations	15684 (779)*
Mean I/σ(I)	12.7 (2.1)*
Completeness (%)	99.9 (100.0)*
Multiplicity	7.0 (7.1)*
CC(1/2)	0.999 (0.953)*
**Refinement**	
Resolution (Å)	35.586 - 2.242
Number of reflections	15564
R_{work}/R_{free}	0.2623/ 0.2912
R_{free} test set, reflections (%)	5.12
No. of non-H atoms	2389
Ligands	2 hemes
R.m.s. ***deviation*** *from ideal geometry*	
Bonds (Å)	0.003
Angles (°)	0. 624
Average B, all atoms (Å^2)	104.46 (range 39.37 – 211.71)
Ramachandran plot	
Favoured regions (%)	96.06
Allowed regions (%)	3.94
Outliers (%)	0
MolProbity clashscore	7.09

* Outer resolution shell

To determine the molecular weights for the BvPgb1.2 WT and Cys86Ala from using size exclusion chromatography, a standard curve was made using standard proteins. The proteins used were colalbumin (75 kDa), carbonic anhydrase (29 kDa), ribonuclease A (13.7 kDa) and aprotinin (6.512 kDa) was used (Cytvia Life Science). The best correlation between the molecular weight and the retention volume was the logarithmic molecular weight as a function of retention volume (Figure S1). By using the corresponding retention volume of BvPgb1.2 WT and Cys86Ala, their molecular weight was estimated.

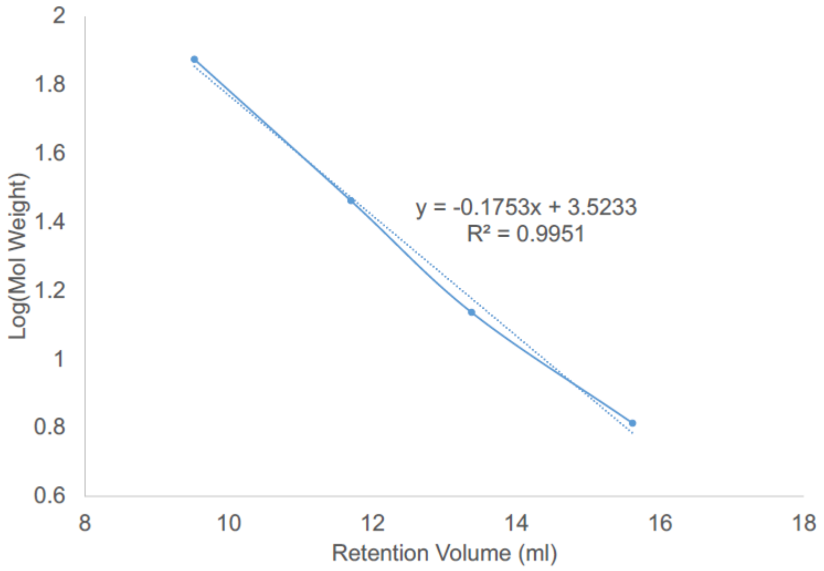

Figure S1. Standard curve from Size Exclusion Chromatography. The standard proteins used were: Colalbumin (75 kDa), carbonic anhydrase (29 kDa), ribonuclease A (13.7 kDa) and aprotinin (6.512 kDa). The logarithm of each molecular weight was plotted as a function of retention volume (ml) for each protein.

In order to estimate the kinetic parameters from Table 1, the decrease in absorbance at 542 and 576 nm was plotted as a function of time. This decay follows an exponential model and can be seen in Figure S2. The wavelength with the greatest decrease in absorbance (576 nm) was used in XPFit (SoftScientific) to estimate the important kinetic parameters from the Exponential Decay Model.

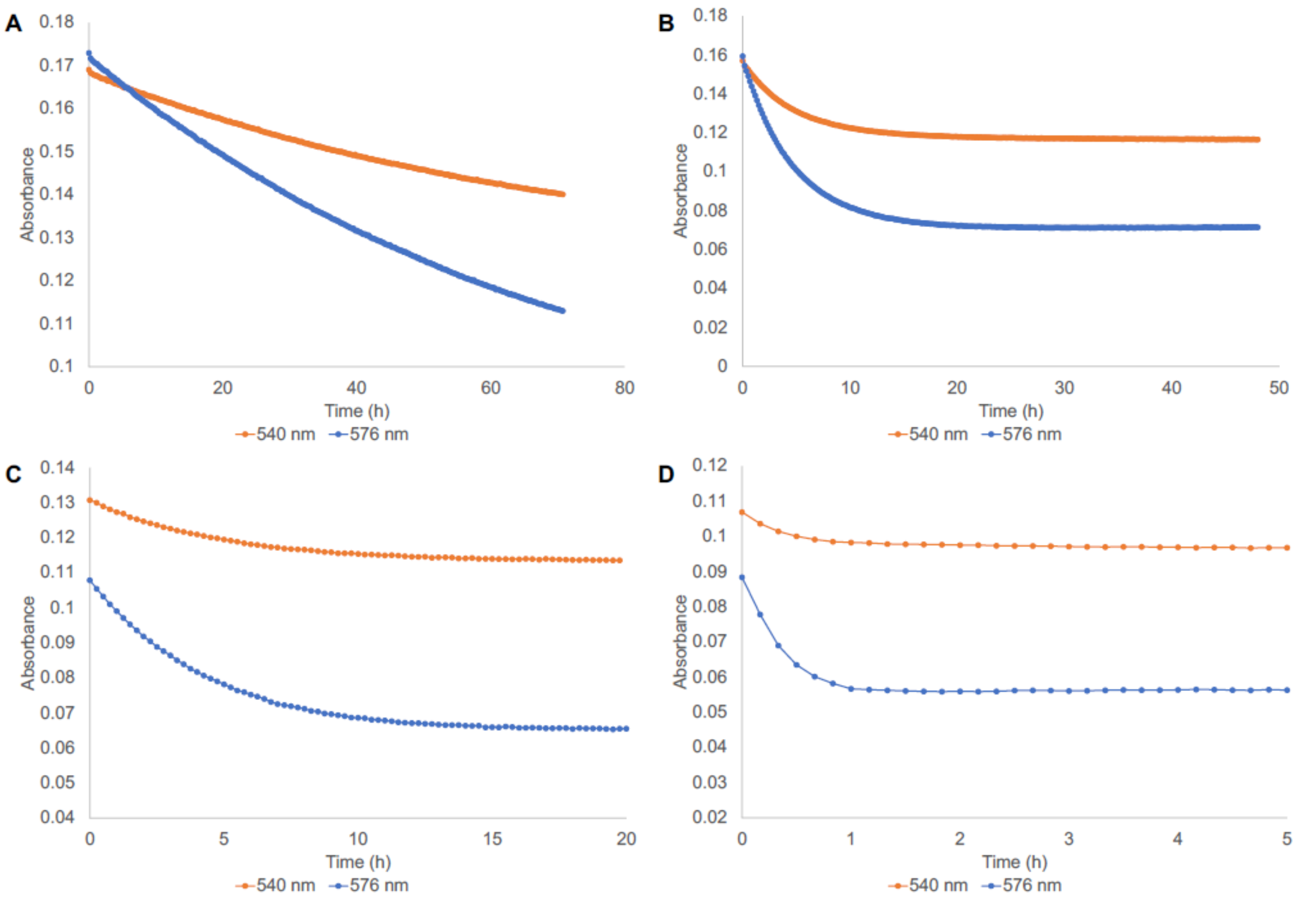

Figure S2. Exponential decay spectra for BvPgb1.2 WT and Cys86Ala mutant at 540 and 576 nm as a function of time. A) WT at 25˚C. B) WT at 37˚C. C) Cys86Ala mutant at 25 ˚C. D) Cys86Ala mutant at 37 ˚C. Data points were taken with 15 min intervals for 72 h at 25 C˚ and with 10 min intervals for 24 h at 37 C˚.

In addition to the heme loss assay seen in Figure 4, the identical assay was redone at 37 ˚C. These results can be seen in Figure S3. Like in the case of 25 ˚C, heme loss was detected for the HbF (positive control), as the Mb spectra emerges at 600 nm. This outcome was not be detected in either WT or Cys86Ala, where the minor change in the first and final spectra was probably due to interference from the added Mb itself and imposed effect of the temperature. The distinctive peak at 600 nm in particular was not be detected.

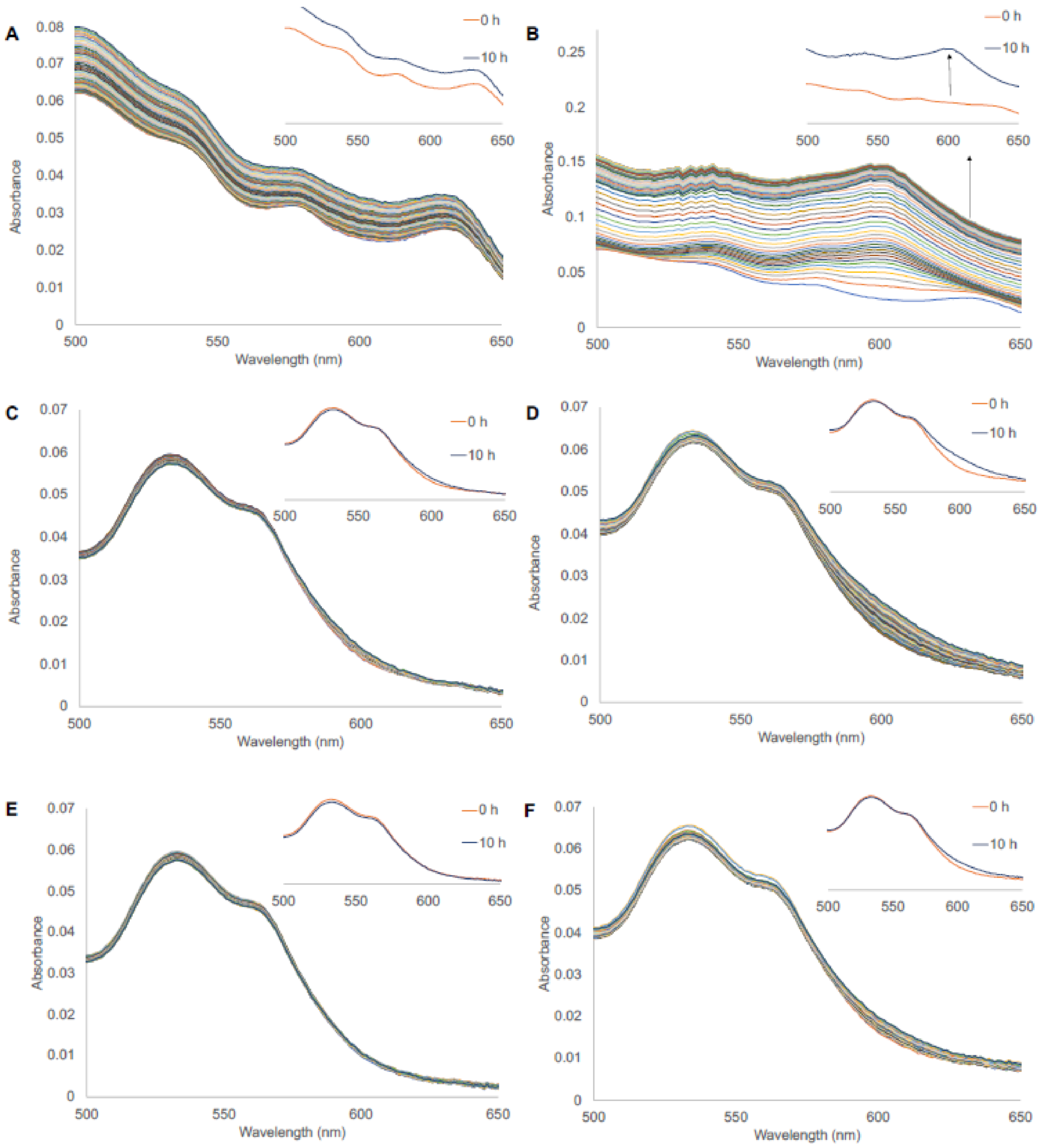

Figure S3. Heme Loss assay using ApoMb as heme-scavenger at 37 ˚C. A) and B): Fetal Hemoglobin (HbF) blank sample and addition of ApoMb, respectively. The blank sample retained the initial spectra after 10 h, while the Mb spectra is detected in the right panel, most predominately seen as increase in absorbance at 600 nm. C) and D): BvPgb1.2 WT blank sample and addition of ApoMb, respectively. E) and F): BvPgb1.2 Cys86Ala blank sample and addition of ApoMb, respectively. All experiments were conducted at 37 ˚C, spectra was measured in 10-minute intervals for 10 h, and the ferric form of the Hbs was used.

To speculate about the electron transfer in/around the heme pocket of BvPgb1.2 WT and why the mutation affected the function of the great extent that it did, the distance between important residues was estimated. In addition to the cysteine, a single tyrosine is present in the amino acid sequence, which is located in close proximity to the heme pocket. The distances between these residues and the heme iron was determined and can be seen in Figure S4. The tyrosine and the cysteine was 9.24 Å and 17.27 Å way from the heme, respectively. Due to the close proximity, it is possible for the these residues to

act like electron doners/acceptors and maintain a sufficient redox environment for the phytoglobin to function and protect it from external factors.

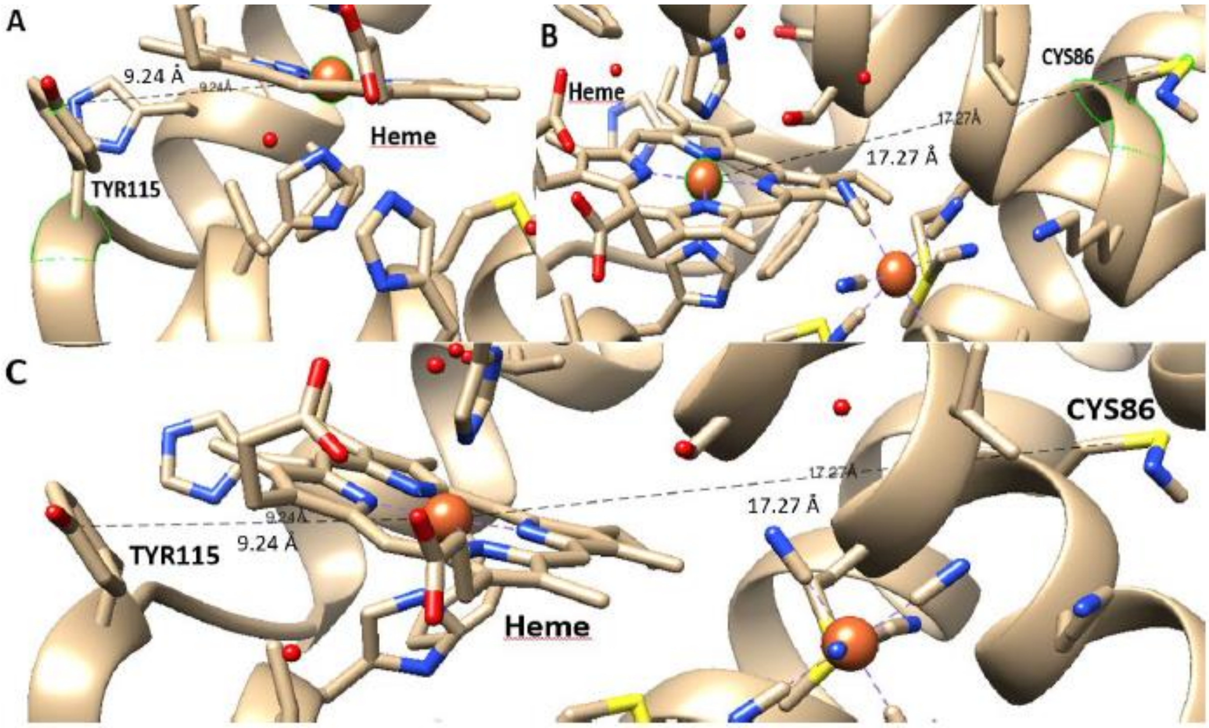

Figure S4. Distance determination between heme and surrounding tyrosine and cysteine residue in BvPgb 1.2 WT. A) Zoom-in picture of single tyrosine of BvPgb 1.2 WT, located 9.2 Å from the heme iron. B) Zoom-in picture of single cysteine of BvPgb 1.2 WT, located 17.3 Å from the heme iron. C) Zoom-in picture of both the tyrosine and cysteine in relation to the heme iron.

In order to determine the thermal stability of BvPgb 1.2 WT and Cys86Ala, a Prometheus NT.48 instrument containing aggregation optics (NanoTemper Technologies) was used. The software determines transitions states during the slow increase in temperature by measuring the absorbance ratio between 350 nm and 330 nm. One example of this can be seen in Figure S5 below, where an example experiment for WT (panel A) and mutant (panel B) are shown. As can be seen in panel A, three states where detected for the WT, while only one transition state were detected for Cys86Ala in panel B. These transitions are detected by measuring the absorbance ration between 350 nm and 330 nm. Even though the precise nature for these transition states can only be speculated in, the difference between the WT and Cys86Ala is evident, pointing to more prominent protein movements/ transitions in the WT.

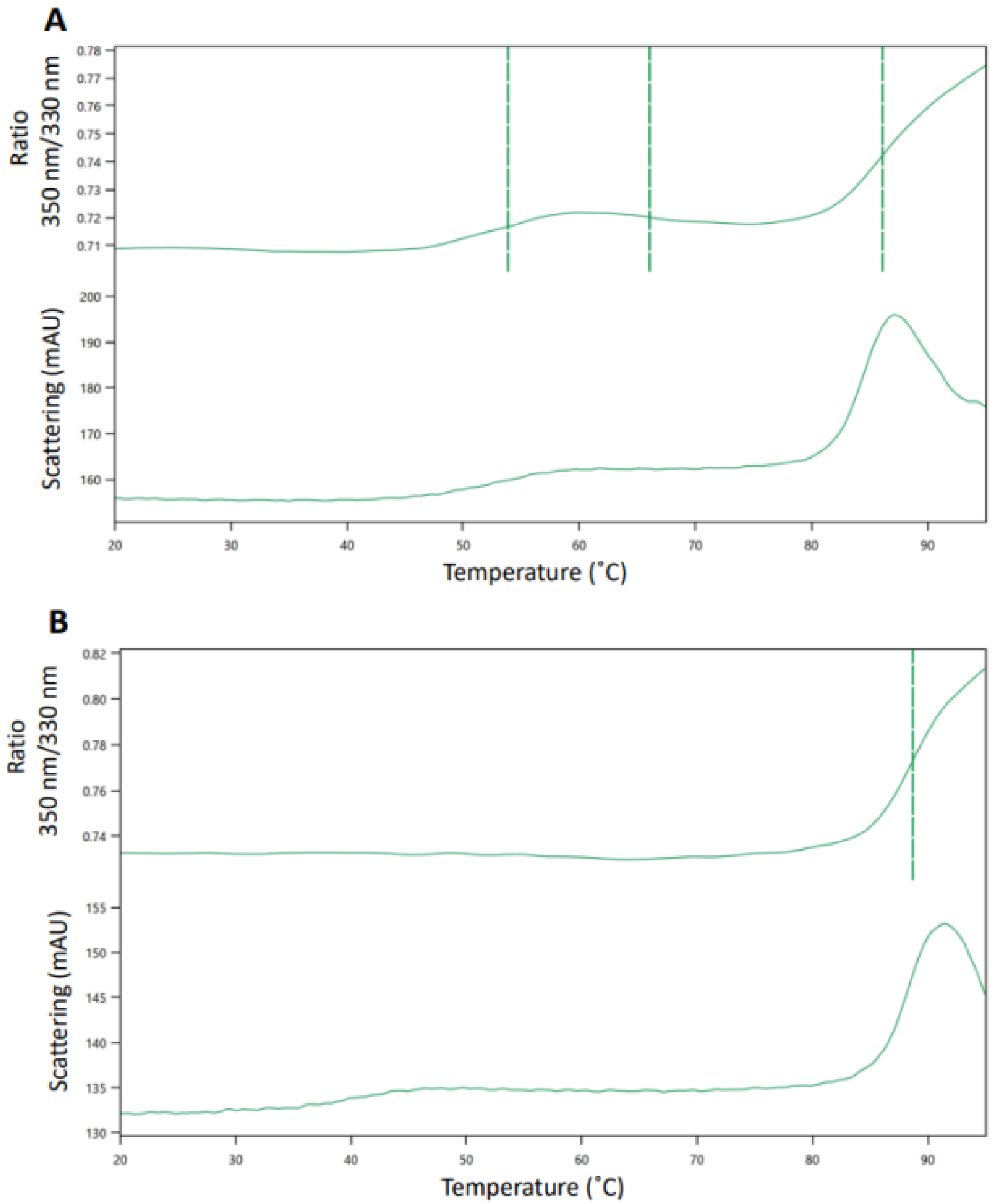

Figure S5. Thermal stability determination using Prometheus NT.48 (NanoTemper Technologies). A) and B): Melting curve for BvPgb 1.2 WT and Cys86Ala, respectively, where the determined transition states are indicated in the ratio section (top panel of A and B). In addition, the absorbance scattering (bottom panel of A and B) is also included. Both experiments were conducted in oxy-form at 25 ˚C during Day 0 of incubation.
